# Supplementary material for: Single-keratinocyte transcriptomic analyses identify different clonal types and proliferative potential mediated by FOXM1 in human epidermal stem cells
Source: Nat Commun. 2021 May 4;12:2505. doi: 10.1038/s41467-021-22779-9 (PMC8097075; doi:10.1038/s41467-021-22779-9)
Supplement: Supplementary file 1 — Supplementary Information [file 41467_2021_22779_MOESM1_ESM.pdf]

Supplementary Information for:

SINGLE-KERATINOCYTE TRANSCRIPTOMIC ANALYSES IDENTIFIES DIFFERENT CLONAL TYPES AND PROLIFERATIVE POTENTIAL MEDIATED BY FOXM1 IN HUMAN EPIDERMAL STEM CELLS

Elena Enzo[1,10], Alessia Secone Seconetti[1,2,10], Mattia Forcato[3], Elena Tenedini[4], Maria Pia Polito[1], Irene Sala[1], Sonia Carulli[2], Roberta Contin[1,9], Clelia Peano[5,6], Enrico Tagliafico[4,7,8], Silvio Bicciato[3], Sergio Bondanza[2] and Michele De Luca[1,✉]

[1]Centre for Regenerative Medicine ''Stefano Ferrari'', University of Modena and Reggio Emilia, Modena, Italy
[2]Holostem Terapie Avanzate, s.r.l, Modena, Italy
[3]Department of Life Sciences, University of Modena and Reggio Emilia, Modena, Italy
[4]Department of Laboratory Medicine and Pathology, Diagnostic hematology and Clinical Genomics Unit, Modena University Hospital, Modena, Italy
[5]Genomic Unit, IRCSS, Humanitas Clinical and Research Center, Rozzano, Milan, Italy
[6]Institute of Genetic and Biomedical Research, UoS Milan, National Research Council, Rozzano, Italy.
[7]Department of Medical and Surgical Sciences, University of Modena and Reggio Emilia, Modena, Italy
[8]Centre for Genome Research, University of Modena and Reggio Emilia, Modena, Italy
[9]Present address: Clinical Sampling & Alliances, AstraZeneca, Cambridge, UK
[10]These authors contributed equally

✉Correspondence: michele.deluca@unimore.it

Table of content

- **Supplementary Data 1:** Genes comprising the Holoclone signature.
- **Supplementary Data 2:** Results of GSEA analysis for bulk and single cell expression data. For each comparison the table reports Normalized Enrichment Score (NES) and False Discovery Rate (FDR).
- **Supplementary Figure 1:** Gene ontology analysis of transcriptomic profile of human epidermal clones.
- **Supplementary Figure 2:** Single cell RNA-seq of human epidermal cells
- **Supplementary Figure 3:** Short term depletion of FOXM1 does not affect keratinocytes proliferation.
- **Supplementary Figure 4:** Analysis of FOXM1 isoforms in human primary keratinocytes.
- **Supplementary Figure 5:** Efficient silencing after siRNA addiction in CNT and FOXM1 overexpressing keratinocytes and immunofluorescence on JEB clones.
- **Supplementary Table 1:** List of antibodies used, source and concentration.
- **Supplementary Table 2:** List of siRNA used with code, type and catalogue number.
- **Supplementary Table 3:** List of taqman probes used for qRT-PCR and catalog number.
- **Supplementary Table 4:** List of primers used with specified name, sequence, reference and application.
- **Source data files** contains source data (.xls) and uncropped blots (.pdf).

SUPPLEMENTARY FIGURE 1

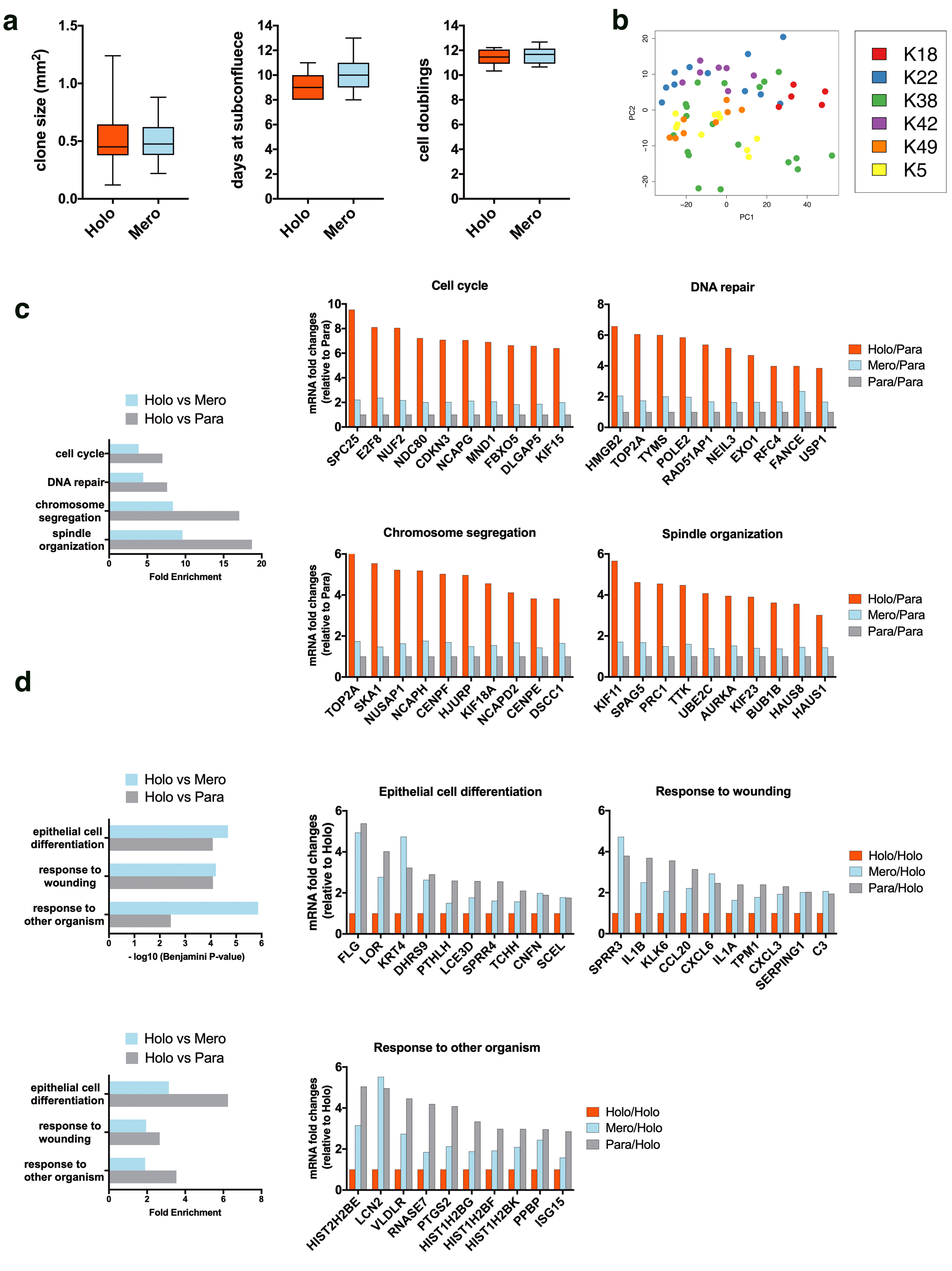

Supplementary Fig. 1: Gene ontology analysis of transcriptomic profile of human epidermal clones

a) Floating bars showing respectively from left to right, size of the clones measured in mm^2 (12 holoclones and 12 meroclones analyzed), days required to reach sub-confluence of the sub-cultivated ¾ of the clones (18 holoclones and 22 meroclones analyzed) and number of cell doublings made by each clone progeny during sub-cultivation (18 holoclones and 22 meroclones analyzed). Data derived from holoclones are identified with red and data derived from meroclones are indicated in light blue. Median and min to max values displayed.

b) Unsupervised principal component analysis (PCA) of holoclone gene expression profiles. Every dot represents a different clone (n=60), different colours represent the different strains.

c) left: Gene ontology (GO) analysis of the genes significantly upregulated (FDR≤5% and fold change≥1.5) in holoclones compared to meroclones (blue bars) and in holoclones compared to paraclones (grey bars). Histograms represent DAVID fold enrichments. Right: expression levels of selected genes of functional categories enriched in genes upregulated in holoclones. Fold changes are measured over paraclones.

d) Gene ontology (GO) analysis of the genes significantly downregulated (FDR≤5% and fold change≤-1.5) in holoclones as compared to meroclones and paraclones. P-values are calculated with one-sided Fisher's Exact test and corrected for multiple tests with Benjamini-Hochberg method. Histograms represent –log10 of the Benjamini score and DAVID fold enrichments. Right: expression levels of selected genes of functional categories enriched in genes downregulated in holoclones. Fold changes are measured over holoclones.

SUPPLEMENTARY FIGURE 2

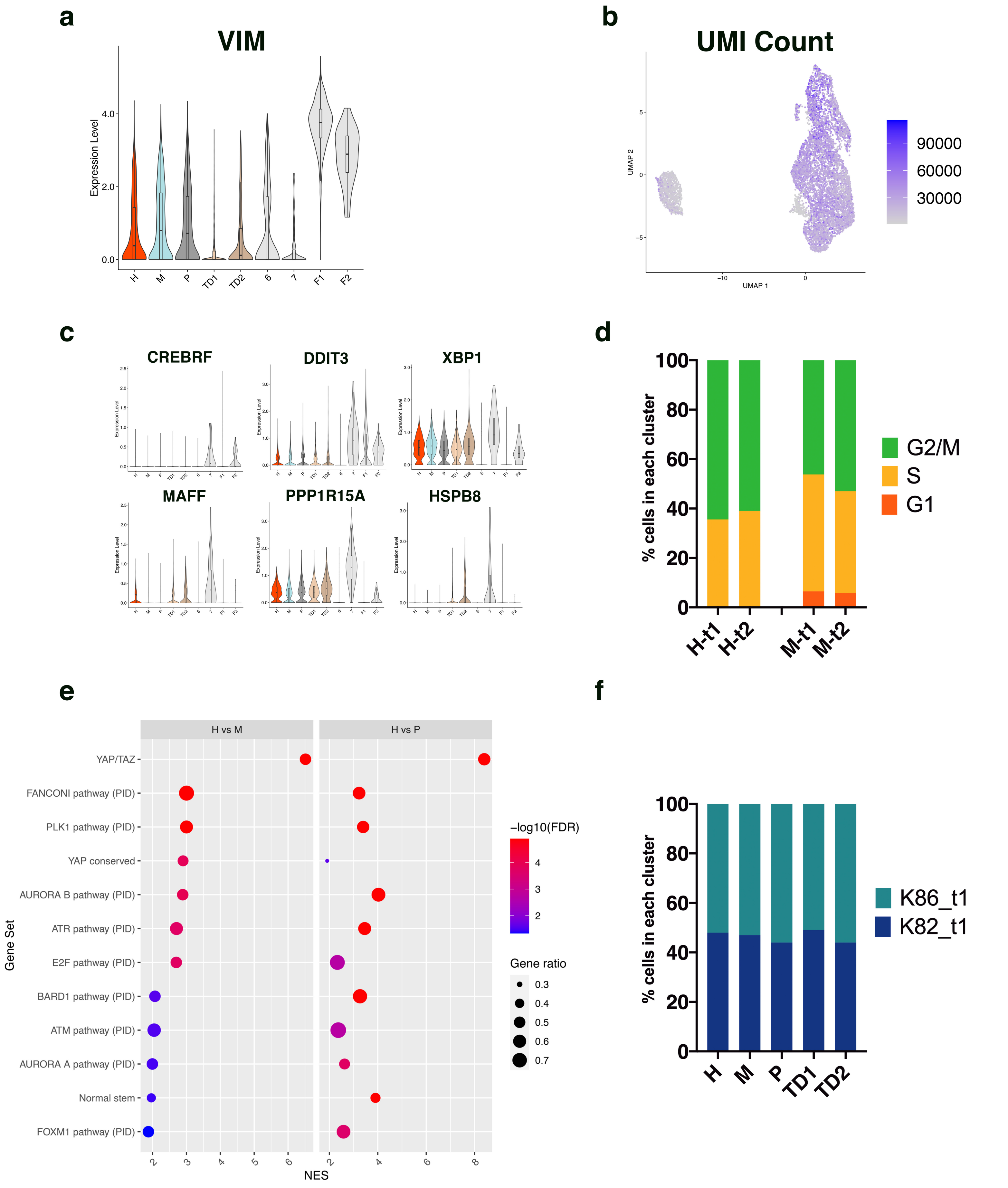

Supplementary Fig. 2: Single cell RNA-seq of human epidermal cells

a) Violin plot showing *VIM* expression in the 9 clusters identified in the integrated scRNA-seq dataset, n=7,345 cells. In boxplots, lines in the middle of boxes correspond to median values. Lower and upper hinges correspond to the first and third quartiles, the upper whisker extends from the hinge to the largest value no further than 1.5 * IQR (inter-quartile range) from the hinge. The lower whisker extends from the hinge to the smallest value at most 1.5 * IQR of the hinge.

b) UMAP plot showing the number of UMI counts identified in each cell.

c) Violin plot showing expression of stress related genes which are highly expressed in cluster 7. n=7,345 cells. In boxplots, lines in the middle of boxes correspond to median values. In boxplots, lines in the middle of boxes correspond to median values. Lower and upper hinges correspond to the first and third quartiles, the upper whisker extends from the hinge to the largest value no further than 1.5 * IQR (inter-quartile range) from the hinge. The lower whisker extends from the hinge to the smallest value at most 1.5 * IQR of the hinge.

d) Percentage of cells in G2/M (green), S (yellow) or G1 (red) phases contained in H and M clusters at the indicated time point

e) Bubble plot showing results of gene set enrichment analysis (GSEA) on scRNA-seq data. The Normalized enrichment score (NES) is indicated on the x-axis; the dot size indicates the fraction of genes contributing to the leading-edge subset within the gene set. Dots are color-coded based on the enrichment FDR. In the image are shown the significant terms upregulated in holoclones both with respect to meroclones and with respect to paraclones.

f) Percenatge of cells contained in each cluster deriving from K82 or K86 at t_1.

SUPPLEMENTARY FIGURE 3

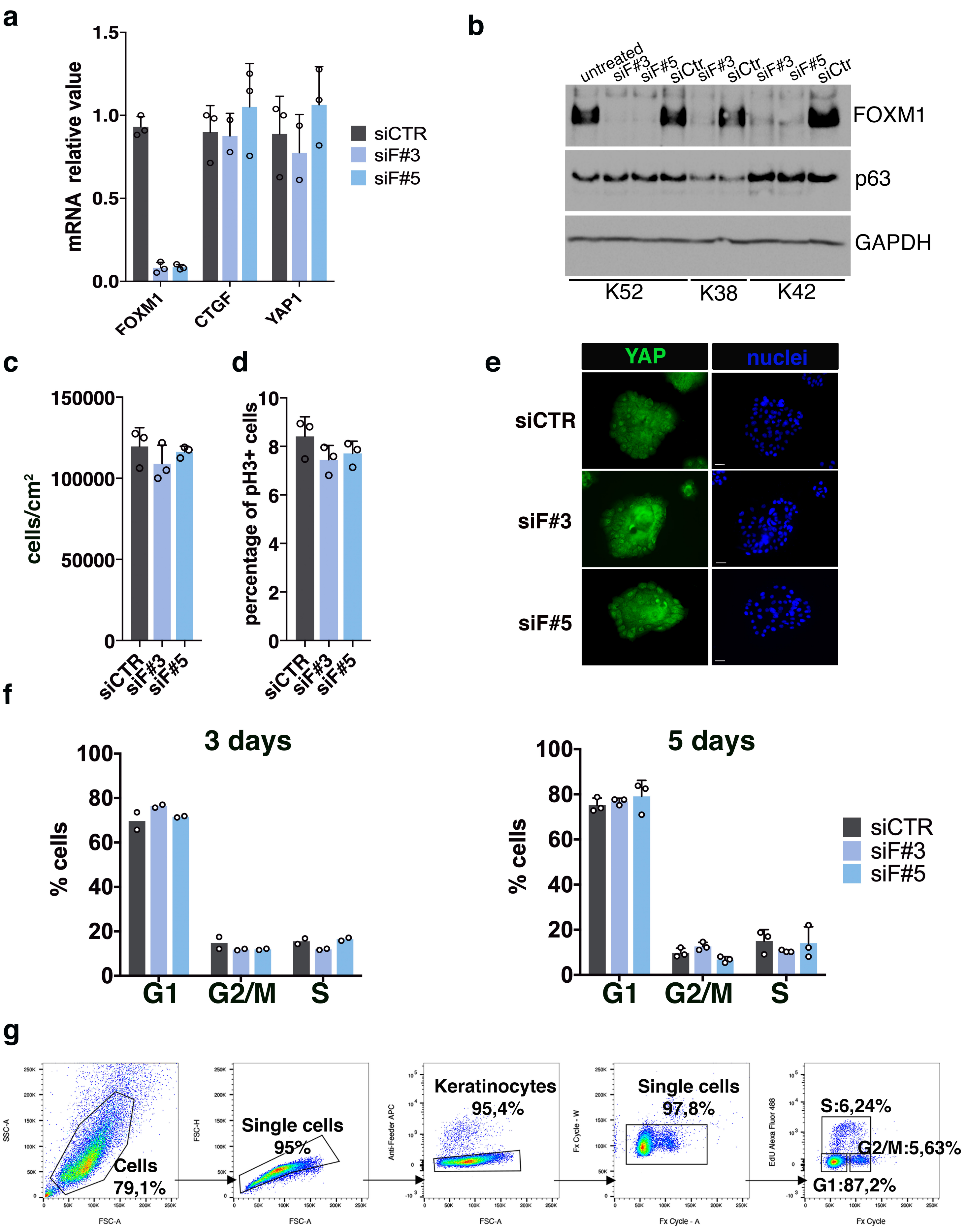

Supplementary Fig. 3: Short term depletion of FOXM1 does not affect keratinocytes proliferation.

a) qRT-PCR on mRNAs obtained from keratinocytes transfected with different siRNA against FOXM1 was used to determine the expression of *FOXM1*, *CTGF* and *YAP*. Expression levels were calculated relative to *GAPDH* and are given relative to siCTR (arbitrarily set to 1). n = 3 independent human primary keratinocyte cultures, indicated with white circles; average and standard deviation displayed.

b) Western analysis of total cell extracts from three independent cultures (K52, K38 and K42) generated by keratinocytes treated with the indicated siRNA stained with indicated antibodies.

c) Amount of cell/cm^2 found after 7 days of cultivation with the indicated siRNA. n = 3 independent human primary keratinocyte cultures, indicated with white circles). Average and standard deviation displayed.

d) Percentage of $pH3^+$ cells among the total amount of cells found after 7 days of cultivation with the indicated siRNA. n = 3 independent human primary keratinocyte cultures, indicated with white circles; Average and standard deviation displayed.

e) Representative image of colonies treated with the indicated siRNA and stained with anti-YAP antibody (green). Nuclei are visualized in blue with DAPI. Scale bar 20 µm.

f) Percentage of cells in G1, G2/M or S phases found after 3 (left) or 5 days (right) of cultivation with the indicated siRNA. 2 (for 3 days) and 3 (for 5 days) biological replicates are indicated by white circles. Average and standard deviation (only for 5 days) displayed.

g) Gating strategy to determine the percentage of cells in each cell cycle phase. The starting cell population was gated based on FSC-A versus SSC-A. Doublets were excluded using FSC-A versus FSC-H and forward scatter versus anti-feeder APC was used to distinguish keratinocytes from 3t3 fibroblasts. Doublets were excluded using FxCycle-A versus FxCycle-W. Fx-cycle VioBlue and EdU Alexa Fluor 488-A were assessed on the keratinocytes population to identify cells in G0/G1 phase, in S phase and in G2/M phase.

SUPPLEMENTARY FIGURE 4

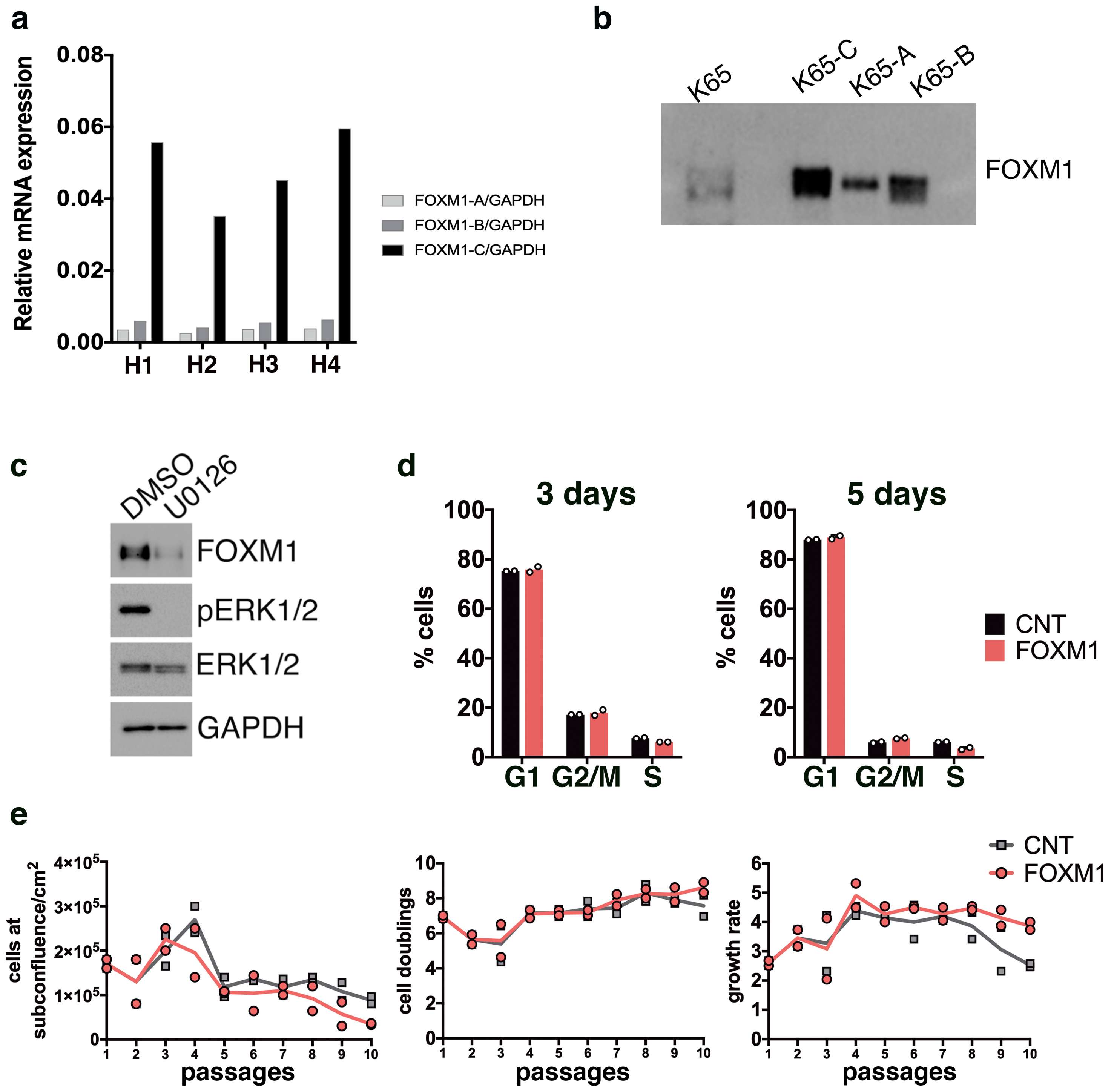

Supplementary Fig. 4: Analysis of FOXM1 isoforms in human primary keratinocytes.

a) qRT-PCR on mRNAs obtained from 4 holoclone-derived mass cultures using primer specific for isoform A, B or C (see Methods). Bars represent expression levels of each holoclone calculated relative to GAPDH.

b) Western analysis of total cell extracts from cultures generated by normal keratinocytes (K65) and keratinocytes overexpressing FOXM1 respectively isoform A, B and C showing that bands present in normal keratinocytes have the same pattern of FOXM1-C (representative images of n = 3).

c) Western analysis of total cell extracts from cultures treated with vehicle (DMSO) or 30μM of U0126 drug showing that endogenous FOXM1 expression decreases when ERK1/2 is not phosphorylated (representative images of n = 3).

d) Percentage of cells in G1, G2/M or S phases found after 3 (left) or 5 days (right) of cultivation of control (CNT) and FOXM1 overexpressing keratinocytes. Bars indicated average value, 2 biological replicates are indicated by white circles**.**

e) Serial cultivation of control (CNT, gray line) and FOXM1-transduced (red line) epidermal cultures. From left to right: number of cells found after trypsinization when cultures arrive at subconfluence; number of cell doublings at each passage and growth rate, calculated as population doublings per passage. Data derived from serial cultivation of two independent strains of human primary keratinocytes. Average values are plotted with line and biological replicates (K38 and K57) are indicated with circles.

SUPPLEMENTARY FIGURE 5

a

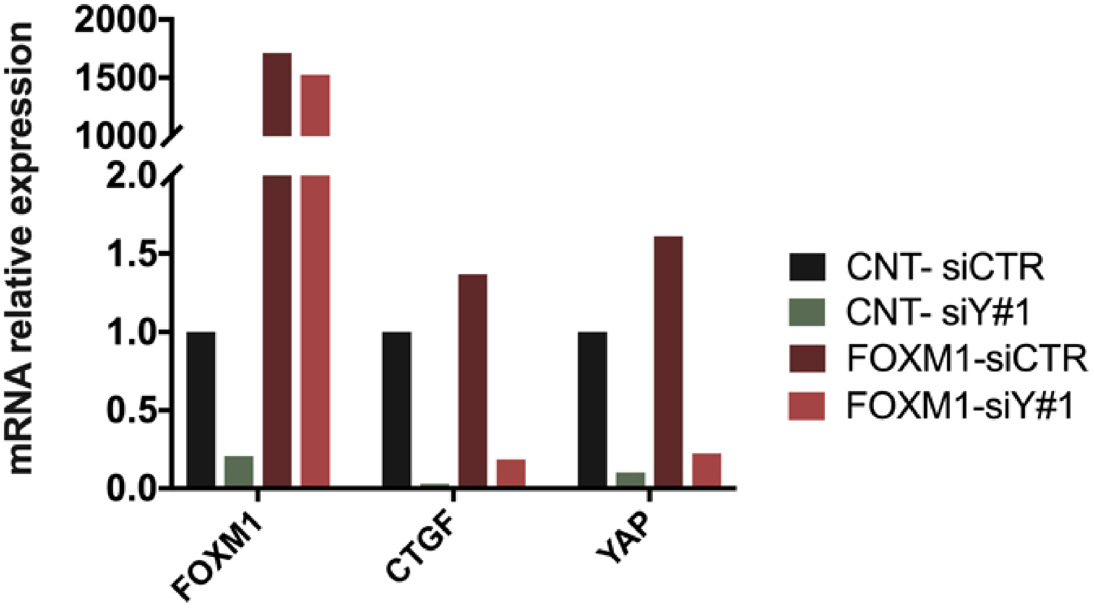

b

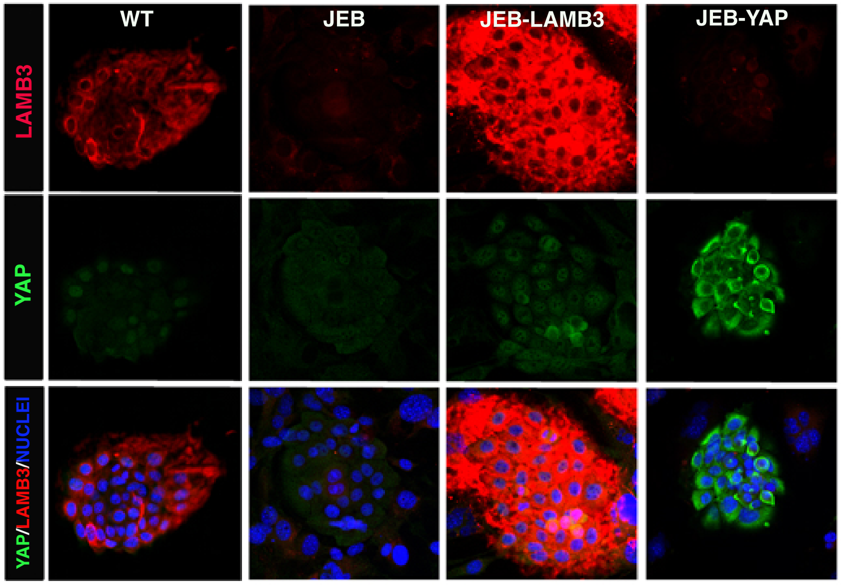

Supplementary Fig. 5: Efficient silencing after siRNA addiction in CNT and FOXM1 overexpressing keratinocytes and immunofluorescence on JEB clones.

a) Related to Figure 5a: qRT-PCR on mRNAs obtained from CNT or FOXM1-transduced keratinocytes transfected with the indicated siRNA was used to determine the expression of *FOXM1*, *CTGF* and *YAP*. Expression levels were calculated relative to *GAPDH* and are given relative to CNT siCTR (arbitrarily set to 1). Bars show value from one representative experiment of 3 biological replicates.

b) Related to Figure 6e: immunofluorescence showing YAP (green) and LAMB3 (red) expression in JEB, LAMB3- and YAP-transduced JEB. DAPI (blue) stains nuclei. Scale bar 20 µm (representative images of n = 3).

Supplementary Table 1: List of antibodies used, source and concentration

Antibody information				Diluition/amount			
**Antibody**	**Company**	**Catalog Number**	**Description**	**Western Blot**	**ChIP**	**IF**	**FACS**
anti-FOXM1	Cell signaling Technology	D3F2B	Rabbit monoclonal	1/1000		1/500	
anti-p63 alfa	Di Iorio et al., 2005	N/A	Rabbit monoclonal	1/2000			
anti-BIRC5	Abcam	ab469	Rabbit polyclonal	1/2000			
anti-GAPDH	Abcam	ab8245	Mouse monoclonal	1/10000			
anti-ITGB1	Abcam	ab52971	Rabbit monoclonal	1/1000			
anti-ITGB4	Santa Cruz Biotechnology	sc-135950	Mouse monoclonal	1/200			
anti-SFN (14-3-3 s)	Abcam	ab14123	Mouse monoclonal	1/200			
anti-YAP1	Millipore	MAB-C203	Mouse monoclonal	1/500			
anti-LAMB3	Santa Cruz Biotechnology	sc-7651	Goat polyclonal	1/700			
anti-FOXM1 (A-11)	Santa Cruz Biotechnology	Sc-271746	Mouse monoclonal			1/500	
anti-Laminin β-3 (6F12)	Patricia Rousselle Laboratory, Lyon	N/A	Mouse monoclonal			1/150000	
anti-YAP	Santa Cruz Biotechnology	sc-101199	Mouse monoclonal			1/500	
anti-MAPK	Cell Signaling Technology	91025	Rabbit polyclonal	1/1000			
anti-phospho MAPK	Cell Signaling Technology	4370L	Rabbit polyclonal	1/1000			
anti-KRT14	Biolegend	905301	Rabbit polyclonal			1/8000	
anti-phospho-Histone H3 (ser10)	Millipore	06-570	Rabbit polyclonal			1/500	
anti-IgG	Abcam	ab171870	Rabbit polyclonal		1/300		
anti-YAP	Cell signaling Technology	D8H1X	Rabbit monoclonal		1/25	1/500	
anti-TEAD4	Abcam	ab58310	Mouse monoclonal		1/300		
Anti-Feeder APC	Milteny biotech	130-102-900	Mouse monoclonal				1/50
Alexa Fluor 488 Azide	Thermo Fisher	C10425B	Mouse monoclonal				1/200
Donkey anti-rabbit IgG HRP	Santa Cruz Biotechnology	sc-2313	secondary antibody	1/1000 - 1/5000			
Donkey anti-mouse IgG HRP	Santa Cruz Biotechnology	sc-2314	secondary antibody	1/10000			
Donkey anti-Mouse IgG (H+L) Alexa Fuor 568	Thermo Fisher Scientific	A10037	secondary antibody			1/2000 - 1/1000	
Donkey anti-Rabbit IgG (H+L) Alexa Fuor 488	Thermo Fisher Scientific	A21206	secondary antibody			1/2000 - 1/1000	

Supplementary Table 2: List of siRNA used with code, type and catalogue number

siRNA name	gene	code	type	cartalog number
siC	negative control		silencer select (Thermo fisher)	4390843
siF#3	FOXM1	s5250	silencer select (Thermo fisher)	4392420
siF#5	FOXM1	s5248	silencer select (Thermo fisher)	4392420
siY#1	YAP1	s536627	silencer select (Thermo fisher)	4392420
siY#2	YAP1	s536628	silencer select (Thermo fisher)	4392420

Supplementary Table 3: List of taqman probes used for qRT-PCR and catalog number

Taqman probes	Catalog Number
ANLN	Hs01122612_m1
AURKB	Hs00945855_g1
CCNA2	Hs00996788_m1
CCNB1	Hs01030099_m1
CKAP2L	Hs00403991_m1
CTGF	Hs01026927_g1
FOXM1	Hs01073586_m1
GAPDH	4332649
HMGB2	Hs01127828_g1
LMNB1	Hs01059210_m1
WDR76	Hs00227706_m1
YAP1	Hs00902712_g1

Supplementary Table 4: List of primers used with specified name, sequence, reference and application

Primer name	sequence	Reference	Application
FoxM1sito-69-FOR	ACCGCACAGCCTTCGAG	adapted from Eisenger-Mathason, 2015	ChIP
FoxM1sito-69-REV	GTTTGAAATTGGCGCCGG	adapted from Eisenger-Mathason, 2015	ChIP
FoxM1sito-652-FOR	GGAAAGAACCTTGTCTGCCA	from Mizuno et al ,2012	ChIP
FoxM1sito-652-REV	AGCCAAGCCTTCGGATATAA	from Mizuno et al ,2012	ChIP
HBB-FOR	GCTTCTGACACAACTGTGTTCACTAGC	Zanconato et al ,2015	ChIP
HBB-REF	CACCAACTTCATCCACGTTCACC	Zanconato et al ,2016	ChIP
CTGF- FOR	TGTGCCAGCTTTTTCAGACG	Zanconato et al ,2017	ChIP
CTGF- REV	TGAGCTGAATGGAGTCCTACACA	Zanconato et al ,2018	ChIP
Foxm1-A FOR	TGGGGAACAGGTGGTGTTTGG	custom made	qRT-PCR
Foxm1-A REV	GCTAGCAGCACTGATAAACAAAG	custom made	qRT-PCR
Foxm1-B FOR	CAGGTGTTTAAGCAGCAGA	custom made	qRT-PCR
Foxm1-C FOR	CAATTGCCCGAGCACTTGGAATCA	custom made	qRT-PCR
Foxm1-BC REV	TCCTCAGCTAGCAGCACCTTG	custom made	qRT-PCR
