# Supplementary material for: Single-keratinocyte transcriptomic analyses identify different clonal types and proliferative potential mediated by FOXM1 in human epidermal stem cells
Source: Nat Commun. 2021 May 4;12:2505. doi: 10.1038/s41467-021-22779-9 (PMC8097075; doi:10.1038/s41467-021-22779-9)
Supplement: Supplementary file 4 — Reporting Summary [file 41467_2021_22779_MOESM4_ESM.pdf]

nature research

Corresponding author(s): De Luca Michele

Last updated by author(s): 20/03/2021

Reporting Summary

Nature Research wishes to improve the reproducibility of the work that we publish. This form provides structure for consistency and transparency in reporting. For further information on Nature Research policies, see our Editorial Policies and the Editorial Policy Checklist.

Statistics

For all statistical analyses, confirm that the following items are present in the figure legend, table legend, main text, or Methods section.

n/a	Confirmed	
☐	☒	The exact sample size (*n*) for each experimental group/condition, given as a discrete number and unit of measurement
☐	☒	A statement on whether measurements were taken from distinct samples or whether the same sample was measured repeatedly
☒	☐	The statistical test(s) used AND whether they are one- or two-sided<br>*Only common tests should be described solely by name; describe more complex techniques in the Methods section.*
☒	☐	A description of all covariates tested
☒	☐	A description of any assumptions or corrections, such as tests of normality and adjustment for multiple comparisons
☐	☒	A full description of the statistical parameters including central tendency (e.g. means) or other basic estimates (e.g. regression coefficient) AND variation (e.g. standard deviation) or associated estimates of uncertainty (e.g. confidence intervals)
☒	☐	For null hypothesis testing, the test statistic (e.g. *F*, *t*, *r*) with confidence intervals, effect sizes, degrees of freedom and *P* value noted<br>*Give P values as exact values whenever suitable.*
☒	☐	For Bayesian analysis, information on the choice of priors and Markov chain Monte Carlo settings
☒	☐	For hierarchical and complex designs, identification of the appropriate level for tests and full reporting of outcomes
☐	☒	Estimates of effect sizes (e.g. Cohen's *d*, Pearson's *r*), indicating how they were calculated

Our web collection on statistics for biologists contains articles on many of the points above.

Software and code

Policy information about availability of computer code

Data collection	qPCR data were acquired and analyzed with RQ Manager 1.2.2 and visualized with Prism 7. Immunofluorescence data were acquired with Zeiss confocal microscope LSM510 and ZEN 2009 software. Western blotting was acquired with ImageLab5.2.1. Flow cytometry data were acquired with BD FACSCanto II.
Data analysis	Microarray data were analyzed with R (version 3.5.0) software.<br>affy (version 1.60.0) R package was used to quantify gene expression.<br>samr (version 3.0) R package was used to perform differential analysis.<br>DAVID (version 6.7) web tool was used for Gene Ontology enrichment analysis.<br>GSEA (version 4.0.3) was used for pathway enrichment analysis.<br>Cell Ranger (version 3.1.0) was used for alignment and expression quantification of scRNA-seq data.<br>Seurat (version 3.1.5) R package was used for single cell data analysis.<br>ggplot (version 3.3.0) R package was used to show the most significantly enriched gene sets in Holoclones.<br>Stats R package was used fo global unsupervised clustering.<br>Monocle3 (version 0.2.1) R package was used for trajectory analysis.<br>BD FACSDiva Software (version 6.1.3) anf FlowJo version 10 were used for flowcytometric analysis.

For manuscripts utilizing custom algorithms or software that are central to the research but not yet described in published literature, software must be made available to editors and reviewers. We strongly encourage code deposition in a community repository (e.g. GitHub). See the Nature Research guidelines for submitting code & software for further information.

Data

Policy information about availability of data

All manuscripts must include a data availability statement. This statement should provide the following information, where applicable:

- Accession codes, unique identifiers, or web links for publicly available datasets
- A list of figures that have associated raw data
- A description of any restrictions on data availability

Microarray and scRNA-seq data are available in Gene Expression Omnibus with accession number GSE155817 GSE155817 [https://www.ncbi.nlm.nih.gov/geo/query/acc.cgi?acc=GSE155817] regarding Figure 1, 2, 3 and Supplementary Fig. 1 and 2. Databased used: Molecular Signature Database: https://www.gsea-msigdb.org/gsea/index.jsp and Brainarray: http://brainarray.mbni.med.umich.edu/Brainarray/Database/CustomCDF/23.0.0/entrezg.asp. Uncropped blots and source data are provided with this paper.

Field-specific reporting

Please select the one below that is the best fit for your research. If you are not sure, read the appropriate sections before making your selection.

- [x] Life sciences
- [ ] Behavioural & social sciences
- [ ] Ecological, evolutionary & environmental sciences

For a reference copy of the document with all sections, see nature.com/documents/nr-reporting-summary-flat.pdf

Life sciences study design

All studies must disclose on these points even when the disclosure is negative.

Sample size	Sample size calculation was not performed. Sample size was chosen based on standards in the field (De Rosa et al., 2019; Meran et al. 2020; Campinoti et al., 2020) and is indicated in figure legends. All key experiments were repeated independently using different human primary keratinocyte cultures derived from different healty donors or patients affected by Junctional Epidermolysis Bullosa<br>- Microarray experiments were performed on 60 clones isolated from 6 different keratinocytes primary cultures<br>- Single-cell RNA-seq was performed on 3.367 and 3.978 derived from two different keratinocytes primary cultures, respectively<br>- Transfections and transductions experiments were performed at least three times, on different keratinocytes primary cultures derived from healthy donors or JEB patient.
Data exclusions	We presented all data obtained
Replication	All experiments were repeated as indicated in figure legends. All qPCR experiments were performed with three technical replicates on the indicated number of different human primary keratinocyte cultures derived from different healty donors. Replicates on experiments with keratinocytes derived from Junctional Epidermolysis Bullosa patient were obtained starting from three independent transductions.
Randomization	Randomization was not relevant. All human primary keratinocytes or biological samples were analysed or treated in the same manner.
Blinding	We applied blinding for microarray and single-cell processing and data analysis. For the other experiments, blinding was not possible but dfferent analysis were conducetd and repeated independently by more experimenters.

Reporting for specific materials, systems and methods

We require information from authors about some types of materials, experimental systems and methods used in many studies. Here, indicate whether each material, system or method listed is relevant to your study. If you are not sure if a list item applies to your research, read the appropriate section before selecting a response.

Materials & experimental systems

n/a	Involved in the study	
☐	☒	Antibodies
☐	☒	Eukaryotic cell lines
☒	☐	Palaeontology and archaeology
☒	☐	Animals and other organisms
☐	☒	Human research participants
☒	☐	Clinical data
☒	☐	Dual use research of concern

Methods

n/a	Involved in the study	
☒	☐	ChIP-seq
☐	☒	Flow cytometry
☒	☐	MRI-based neuroimaging

Antibodies

Antibodies used	Primary antibodies:

anti-FOXM1 rabbit monoclonal, Cell signaling Technology D3F2B
anti-p63 alfa rabbit monoclonal, Di Iorio et al., 2005 N/A
anti-BIRC5 rabbit polyclonal, Abcam ab469
anti-GAPDH mouse monoclonal, Abcam ab8245
anti-ITGB1 rabbit monoclonal, Abcam ab52971
anti-ITGB4 mouse monoclonal, Santa Cruz Biotechnology sc-135950
anti-SFN (14-3-3 s) mouse monoclonal, Abcam ab14123
anti-YAP1 mouse monoclonal, Millipore MAB-C203
anti-LAMB3 goat polyclonal, Santa Cruz Biotechnology sc-7651
anti-Laminin-β-3 (6F12) mouse monoclonal, from Patricia Rousselle Laboratory, Lyon
anti-MAPK Cell Signaling Technology 91025
anti-phospho-MAPK Cell signaling Technology 4370L
anti-YAP mouse monoclonal, Santa Cruz Biotechnology sc-101199
anti-KRT14 rabbit polyclonal, Biolegend 905301
anti-phospho-Histone H3 (ser10) rabbit polyclonal, Millipore 06-570
anti-IgG rabbit polyclonal, Abcam, ab171870
anti-YAP rabbit monoclonal, Cell signaling Technology D8H1X
anti-TEAD4 mouse monoclonal, Abcam ab58310
anti-FOXM1 (A-11) mouse monoclonal, Santa Cruz Biotechnology sc-271746
Anti-Feeder APC mouse monoclonal, Milteny biotech 130-102-900

Secondary antibodies:
Donkey anti-rabbit igG HRP Santa Cruz Biotechnology sc-2313
Donkey anti-mouse igG HRP Santa Cruz Biotechnology sc-2314
Donkey anti-Mouse IgG (H+L) Secondary Antibody, Alexa Fuor 568 Thermo Fisher Scientific A10037
Donkey anti-Rabbit IgG (H+L) Secondary Antibody, Alexa Fuor 488 Thermo Fisher Scientific A21206
Alexa Fluor 488 Azide mouse monoclonal, Thermo Fisher C10425B

Validation

1. anti-FOXM1 Cell signaling Technology D3F2B (https://www.cellsignal.com/products/primary-antibodies/foxm1-d3f2b-rabbit-mab/20459): validated by manufacturer to recognize endogenous levels in HT-29, DLD-1 and MCF-10A cells for immunofluorescence, western blot, immunohistochemistry and chromatin immunoprecipitation applications. Validated by authors with specific siRNA against FOXM1 (Supplementary Figure 3b), shRNA (Figure 4b) or after overexpression (Figure 4e).
2. anti-p63 alfa was produced by PRIMM (Milan) by immunization of rabbits with a synthetic peptide specific for p63-α, NH2, DFNFDMDARRNKQQRIKEEGECOOH, comprising the C terminus postSAM domain of p63α. It was validated in Di Iorio et al, 2005 (https://www.pnas.org/content/102/27/9523.long), De Rosa et al, 2019 (https://www.sciencedirect.com/science/article/pii/S2211124719305285?via%3Dihub)
3. anti-BIRC5 Abcam ab469 (https://www.abcam.com/survivin-antibody-ab469.html): validated by provider in lysates of different types of cells (Hela, A431, Jurkat and HEK293) for immunofluorescence, western blot and immunohistochemistry applications. Antibody was also internally validated in De Rosa et al, 2019 (https://www.sciencedirect.com/science/article/pii/S2211124719305285?via%3Dihub)
4. anti-GAPDH Abcam ab8245 (https://www.abcam.com/gapdh-antibody-6c5-loading-control-ab8245.html): validated by provider in lysates of different types of cells (HeLa nuclear, HeLa whole cell lysate, A431, Jurkat ,HEK-293) for Western Blot and Immunofluorescence applications.
5. anti-ITGB1 Abcam ab52971 (https://www.abcam.com/integrin-beta-1-antibody-ep1041y-ab52971.html): validated by provider with wild-type HAP1 cell lysate, integrin beta 1 knockout HAP1 cell lysate and in human lung tissue for Western Blot and immunohistochemistry applications. Antibody was also internally validated in De Rosa et al, 2019 (https://www.sciencedirect.com/science/article/pii/S2211124719305285?via%3Dihub).
6. anti-ITGB4 Santa Cruz Biotechnology sc-135950: validated by manufacturer in A-341 whole cell lysate for Western Blot, immunofluorescence and immunoprecipitation. It was also validated in in De Rosa et al, 2019 (https://www.sciencedirect.com/science/article/pii/S2211124719305285?via%3Dihub).
7. anti-SFN (14-3-3 s) Abcam ab14123 (currently not available, suggested replacement are ab77187 or ab193667): validated by provider in lysates of wild-type HeLa cells, SFN knockout HeLa cells and A431 cells for Western Blot, immunoprecipitation and immunohistochemistry applications.
8. anti-YAP1 Millipore MAB-C203: validated by manufacturer in HeLa cell lysates for Western Blot and ICC apllications and in De Rosa et al 2019 (https://www.sciencedirect.com/science/article/pii/S2211124719305285?via%3Dihub). Validated by authors with specific siRNA against YAP in Figure 5C and after overexpression in Figure 6C.
9. anti-LAMB3 Santa Cruz Biotechnology sc-7651 (currently replaced by sc-133178): validated by manufacturer in NHK, A431n Hacat cells and in De Rosa et. al, 2019 (https://www.sciencedirect.com/science/article/pii/S2211124719305285?via%3Dihub). Validated also by authors in patients affected by Lamb3 gene mutation and after Lamb3 mediated gene therapy in Figure 6b.
10. anti-FOXM1 (A-11) Santa Cruz Biotechnology (https://www.scbt.com/it/p/foxm1-antibody-a-11) is a mouse monoclonal antibody specific for an epitope mapping between amino acids 737-763 at the C-terminus of FOXM1 of human origin. Validated by the provider for western blot and immunofluorescence in MCF7 and SRJH30, U-251-MG and NTERA-. Validated by the authors for western blot and immunofluorescence in experiments with specific siRNA against FOXM1 and after overepression of FOXM1.
11. anti-Laminin β-3 (6F12) Patricia Rousselle Laboratory, Lyon N/A: validated in Marinkovich et al, 1992 (https://rupress.org/jcb/article/119/3/695/14523/The-dermal-epidermal-junction-of-human-skin), Hirsch et. al, 2017 (https://www.nature.com/articles/nature24487) and De Rosa et. al 2019 (https://www.sciencedirect.com/science/article/pii/S2211124719305285?via%3Dihub).
12. anti-YAP Santa Cruz Biotechnology sc-101199 clone 63.7 (https://www.scbt.com/it/p/yap-antibody-63-7): validated by manufacturer in Hela nuclear extract for western Blot, immunohistochemistry, immunofluorescence, immunoprecipitation and ELISA and in Dupont et al, 2010, De Rosa et al, 2019 (https://www.sciencedirect.com/science/article/pii/S2211124719305285?via%3Dihub).

13. anti-MAPK Cell Signaling Technology, 91025 (https://www.cellsignal.com/products/primary-antibodies/p44-42-mapk-erk1-2-antibody/9102): validated by the manufacturer and produced by immunizing animals with a synthetic peptide corresponding to a sequence in the C-terminus of rat p44 MAP Kinase. It was validated for Western Blot, immunofluorescence and immunohistochemistry applications.
14. anti-phospho-MAPK Cell Signaling Technology, 4370L (https://www.cellsignal.com/products/primary-antibodies/phospho-p44-42-mapk-erk1-2-thr202-tyr204-d13-14-4e-xp-rabbit-mab/4370): validated by the manufacturer and produced by immunizing animals with a synthetic phosphopeptide corresponding to residues surrounding Thr202/Tyr204 of human p44 MAP kinase. It was validated for Western Blot, immunohistochemistry, immunofluorescence, fluorescence and immunoprecipitation apllications.
15. anti-KRT14 Biolegend 905301 (https://www.biolegend.com/en-us/products/keratin-14-polyclonal-antibody-purified-10953?GroupID=GROUP26): validated by manifacturer for IHC on skin samples. Validated by the authors for immunofluorescence in a colture with human keratinocytes (positive signal) and fibroblasts (negative signal) in Figure 6e.
16. anti-phospho-Histone H3 (ser10) Millipore 06-570 (https://www.merckmillipore.com/IT/it/product/Anti-phospho-Histone-H3-Ser10-Antibody-Mitosis-Marker,MM_NF-06-570): Evaluated by provider by Western Blot in Colcemid treated HeLa acid extract.
17. anti-IgG Abcam ab171870 (https://www.abcam.com/rabbit-igg-polyclonal-isotype-control-chip-grade-ab171870.html): Validated by provider in Western Blot in human tissuse lives lisate, mouse liver, mouse rat, hela, jurkat and HEK293 and ChIP with X-cHIP abcam Protocol in Hela cells
18. anti-YAP Cell signaling Technology D8H1X (https://www.cellsignal.com/products/primary-antibodies/yap-d8h1x-xp-rabbit-mab/14074): validated by provider in extracts from control HeLa cells and YAP knockout HeLa cells for Western Blot, fluorescence, immunofluorescence, immunohistochemistry, immunoprecipitation, chromatin immunoprecipitation and Cut&Run appllications. Validated by authors in gain and loss of YAP experiments
19. anti-TEAD4 Abcam ab58310 (https://www.abcam.com/tead4-antibody-ab58310.html): validated by provider with wild-type HAP1 whole cell lysate and TEAD4 knockout HAP1 whole cell lysate.

Secondary antibodies:
1. Donkey anti-rabbit IgG HRP Santa Cruz Biotechnology sc-2313 (https://www.scbt.com/p/donkey-anti-rabbit-igg-hrp?bvrrp=Main_Site-en_US/reviews/product/2/2313.htm): 88 citations
2. Donkey anti-mouse IgG HRP Santa Cruz Biotechnology sc-2314 (https://www.scbt.com/p/donkey-anti-mouse-igg-hrp?requestFrom=search): 58 citations
3. Donkey anti-Mouse IgG (H+L) Secondary Antibody, Alexa Fuor 568 Thermo Fisher Scientific A10037 (https://www.thermofisher.com/antibody/product/Donkey-anti-Mouse-IgG-H-L-Highly-Cross-Adsorbed-Secondary-Antibody-Polyclonal/A10037): 387 citations
4. Donkey anti-Rabbit IgG (H+L) Secondary Antibody, Alexa Fuor 488 Thermo Fisher Scientific A21206 (https://www.thermofisher.com/antibody/product/Donkey-anti-Rabbit-IgG-H-L-Highly-Cross-Adsorbed-Secondary-Antibody-Polyclonal/A-21206): 3006 citations
5. Alexa Fluor 488 Azide mouse monoclonal, Thermo Fisher C10425B (https://www.thermofisher.com/order/catalog/product/C10420#/C10420): kit 28 citations

Eukaryotic cell lines

Policy information about cell lines

Cell line source(s)	- Mouse 3T3-J2 cells were a gift from Prof. Howard Green, Harvard Medical School (Boston, MA, USA). Reference: Rheinwald and Green, 1975. Cell; Barrandon et Green, PNAS, 1987; Gallico et al., NEJM, 1984.<br>-MFG-LAMB3-packaging cell line: a retroviral vector expressing the full-length 3.6-kb LAMB3 cDNA under the control of the MLV LTR was constructed by cloning a 3.6-kb of LAMB3 cDNA (Gene Bank Accession #Q13751) into MFG-backbone (Markowitz, D. et al., Virology 167, 400-406 (1988)). The Am12-MGFLAMB3 producer cell lines were generated by transinfection in the amphotropic Gp+envAm12 packaging cell line (Mathor, M. B. et al., Proc Natl Acad Sci U S A 93, 10371-10376, doi:10.1073/pnas.93.19.10371 (1996))<br>- HEK293T cell line: provided by ATCC (Cat# CRL-3216) https://www.lgcstandards-atcc.org/products/all/CRL-3216.aspx#generalinformation
Authentication	- A clinical grade 3T3-J2 cell bank was established under GMP standards by a qualified contractor (EUFETS, GmbH, Idar-Oberstein, Germany), according to the ICH guidelines. GMP-certified 3T3-J2 cells have been authorized for clinical use by national and European regulatory authorities.<br>- MFG-LAMB3-packaging cell line: the entire cDNA of LAMB3 was fully sequenced. A master cell bank of a high-titer packaging clone (GP+envAm12-LAMB3 cells) was produced and obtained made under GMP standards by a qualified contractor (Molmed S.p.A, Milan, Italy) according to the ICH guidelines. All certifications, quality and safety tests (including detection of viruses and other micro-organisms both in vitro and in vivo) were performed under GMP standards.<br>- HEK293T: Cells are kept in liquid nitrogen in labelled amps and located according to a specific listing. Morfology and biological behaviour in accordance with guidelines from ATCC
Mycoplasma contamination	All cell lines were tested and resulted negative for mycoplasma.
Commonly misidentified lines<br>(See ICLAC register)	No commonly misidentified cell lines were used in this study.

Human research participants

Policy information about studies involving human research participants

Population characteristics	Human healthy skin biopsies were obtained as anonymized surgical waste, typically from abdominoplasty or mammoplasty after informed consent. Primary EB keratinocyte were obtained from a 1cm2 biopsies taken (after informed consent) from a patient suffering from LAMB3-dependent Junctional Epidermolysis Bullosa (JEB) at the age of 1 month.
Recruitment	No selection was applied to human samples.
Ethics oversight	Comitato Etico dell'Area Vasta Emilia Nord number 178/09 for healthy donors and Comitato Etico dell'Area Vasta Emilia Nord number 124/2016 for patient affected by Junctional Epidermolysis Bullosa

Note that full information on the approval of the study protocol must also be provided in the manuscript.

Flow Cytometry

Plots

Confirm that:

- [x] The axis labels state the marker and fluorochrome used (e.g. CD4-FITC).
- [x] The axis scales are clearly visible. Include numbers along axes only for bottom left plot of group (a 'group' is an analysis of identical markers).
- [x] All plots are contour plots with outliers or pseudocolor plots.
- [x] A numerical value for number of cells or percentage (with statistics) is provided.

Methodology

Sample preparation	Healthy donors-derived human primary keratinocytes were used for flow cytometry analysis. Cell cycle analysis was performed using Click-iT(TM) EdU Flow Cytometry Assay Kit by Invitrogen according to the manufacturer protocol. In brief, EdU was added to the cells as per dilution 1:1000, after 2 hours cells were harvested and stained for 3t3-feeder cells. Cells were then fixed, permeabilized, incubated with Click-iT reaction cocktail and, at last, stained with FxCycleTMViolet. Stained cells were analyzed with BD FACSCanto II.
Instrument	BD FACSCanto II
Software	BD FACSDiva Software v6.1.3, FlowJo v.10
Cell population abundance	Our cell population contained human keratinocytes and 3t3 fibroblasts. Keratinocytes were sorted based on the gating strategy below.
Gating strategy	The starting cell population was gated based on FSC-A versus SSC-A. Doublets were excluded using FSC-A versus FSC-H and forward scatter versus anti-feeder APC was used to distinguish keratinocytes from 3t3 fibroblasts. Doublets were excluded using FxCycle-A versus FxCycle-W. Fx-cycle VioBlue and EdU Alexa Fluor 488-A were assessed on the keratinocytes population to identify cells in G0/G1 phase, in S phase and in G2/M phases. An example of the gating strategy is represented in Supplementary Figure 3g.

- [x] Tick this box to confirm that a figure exemplifying the gating strategy is provided in the Supplementary Information.
