# Supplementary material for: Single-keratinocyte transcriptomic analyses identify different clonal types and proliferative potential mediated by FOXM1 in human epidermal stem cells
Source: Nat Commun. 2021 May 4;12:2505. doi: 10.1038/s41467-021-22779-9 (PMC8097075; doi:10.1038/s41467-021-22779-9)
Supplement: Supplementary file 5 — Description of Additional Supplementary Files [file 41467_2021_22779_MOESM5_ESM.pdf]

Title: Supplementary Data 1:
Description: *Genes comprising the Holoclone signature*

Title: Supplementary Data 2:
Description: *Results of GSEA analysis for bulk and single cell expression data. For each comparison the table reports Normalized Enrichment Score (NES) and False Discovery Rate (FDR).*
