# Supplementary material for: Single-keratinocyte transcriptomic analyses identify different clonal types and proliferative potential mediated by FOXM1 in human epidermal stem cells
Source: Nat Commun. 2021 May 4;12:2505. doi: 10.1038/s41467-021-22779-9 (PMC8097075; doi:10.1038/s41467-021-22779-9)
Supplement: Supplementary file 7 — Source Data [file 41467_2021_22779_MOESM7_ESM.zip › 11_uncroppedBlots-NCOMMS-20-44258A_rev.pdf]

Supplementary Fig 3b

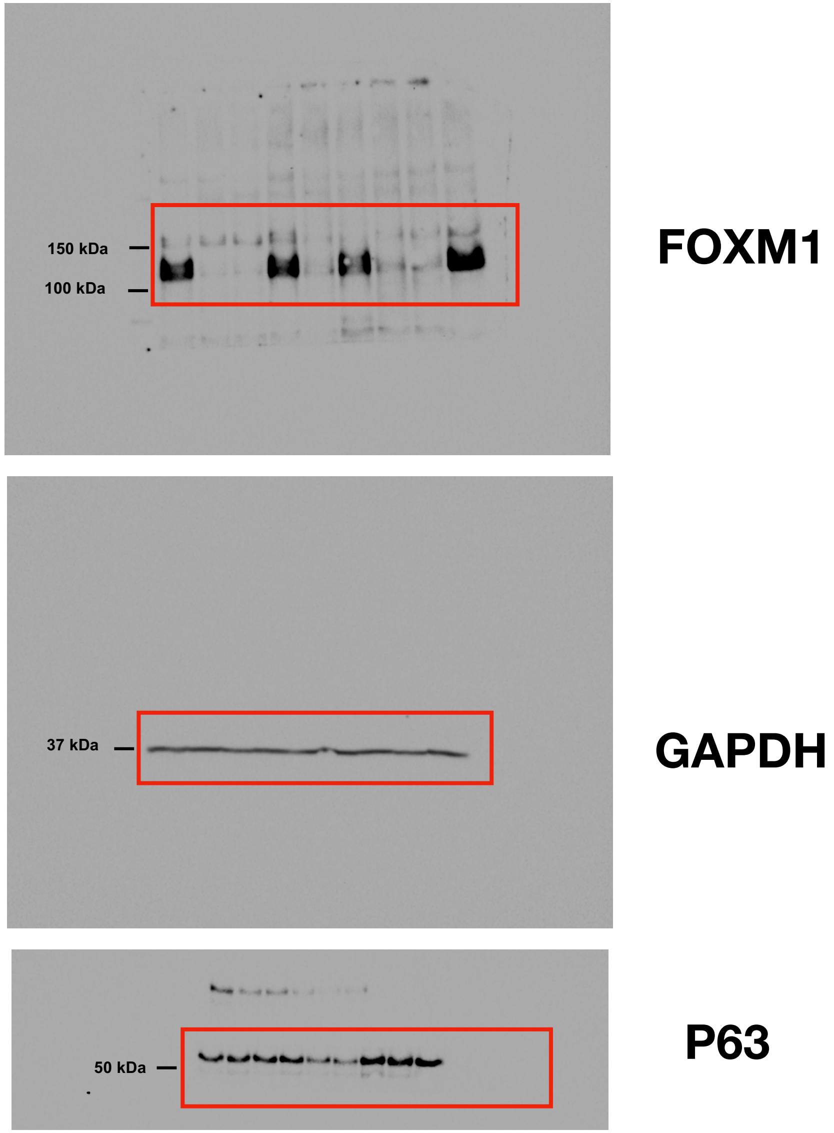

Fig 4a

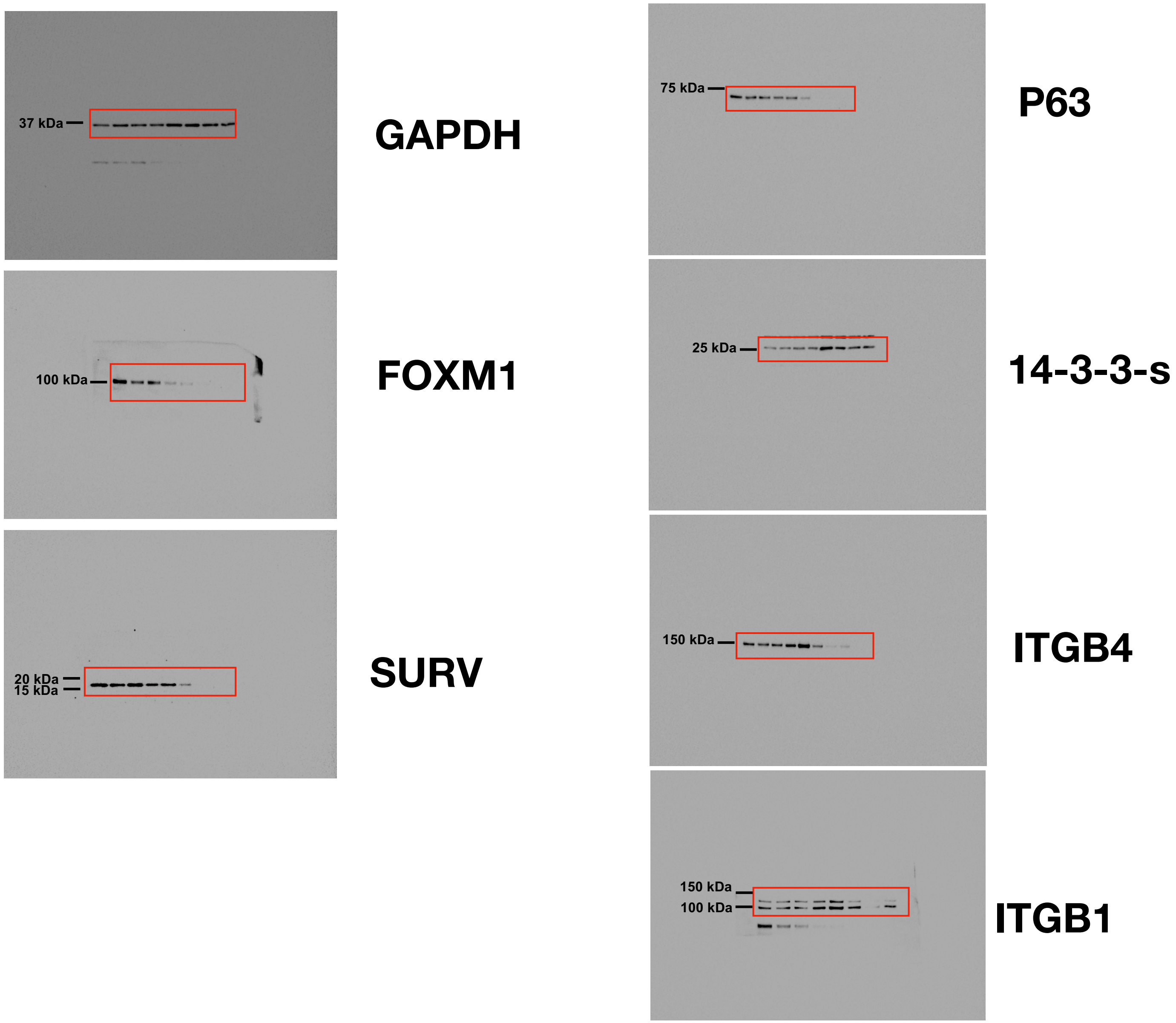

37 kDa
GAPDH
100 kDa
FOXM1
20 kDa
15 kDa
SURV
75 kDa
P63
25 kDa
14-3-3-s
150 kDa
ITGB4
150 kDa
100 kDa
ITGB1

Fig 4b

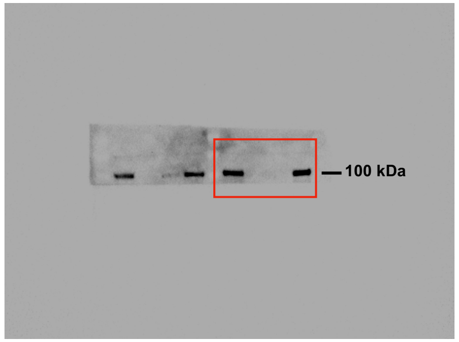

FOXM1

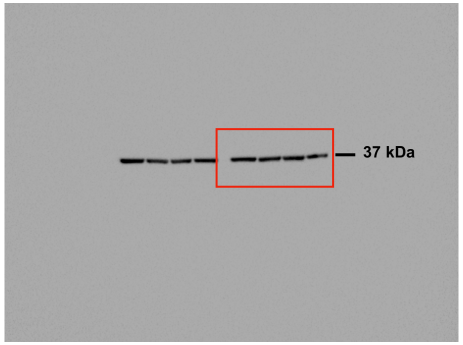

GAPDH

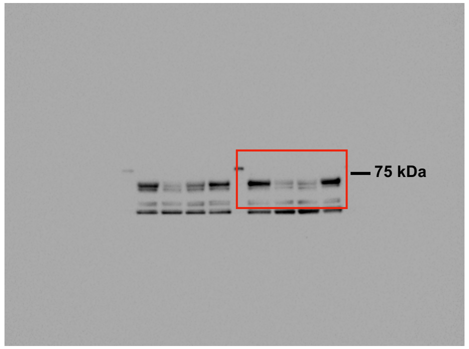

P63

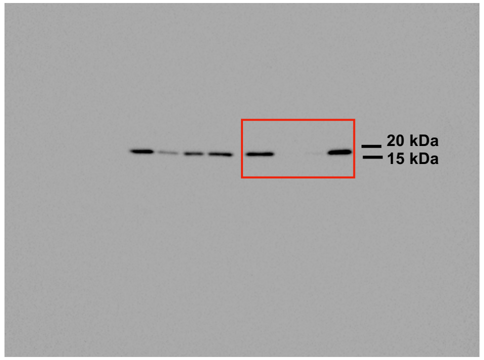

SURV

Fig 4e

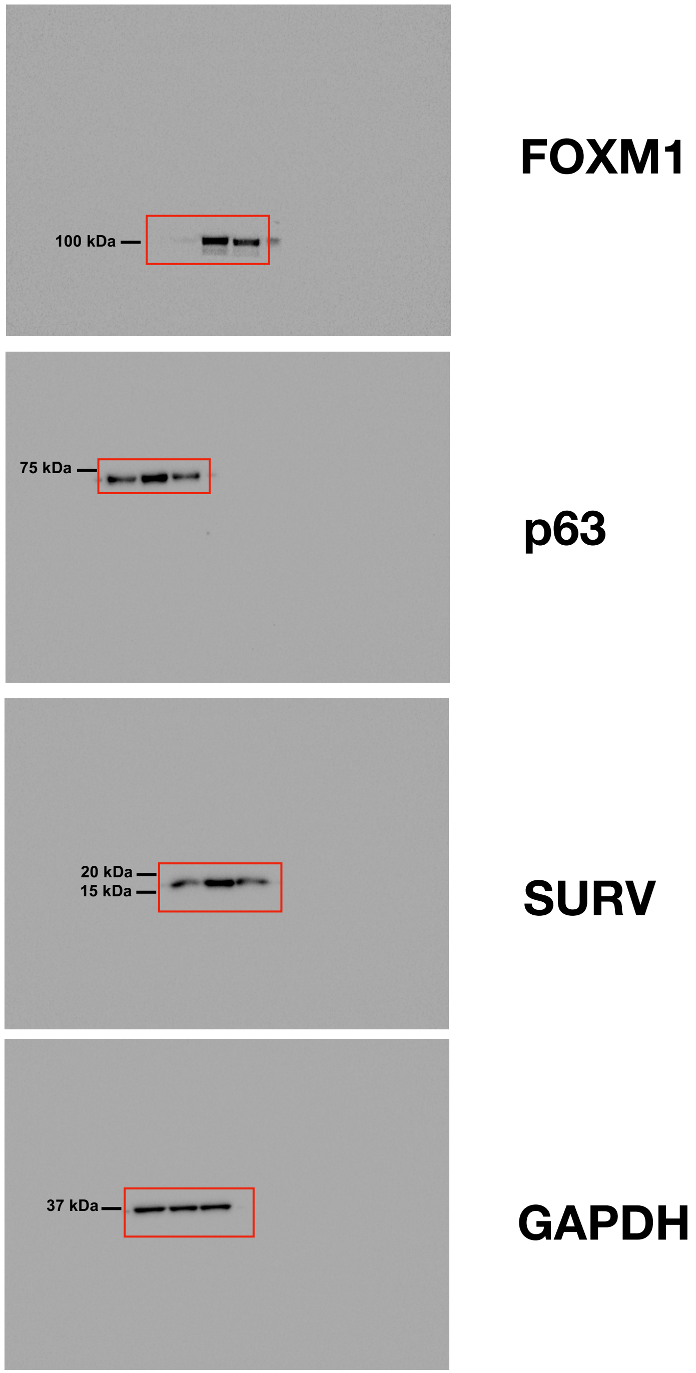

Supplementary Fig 4c

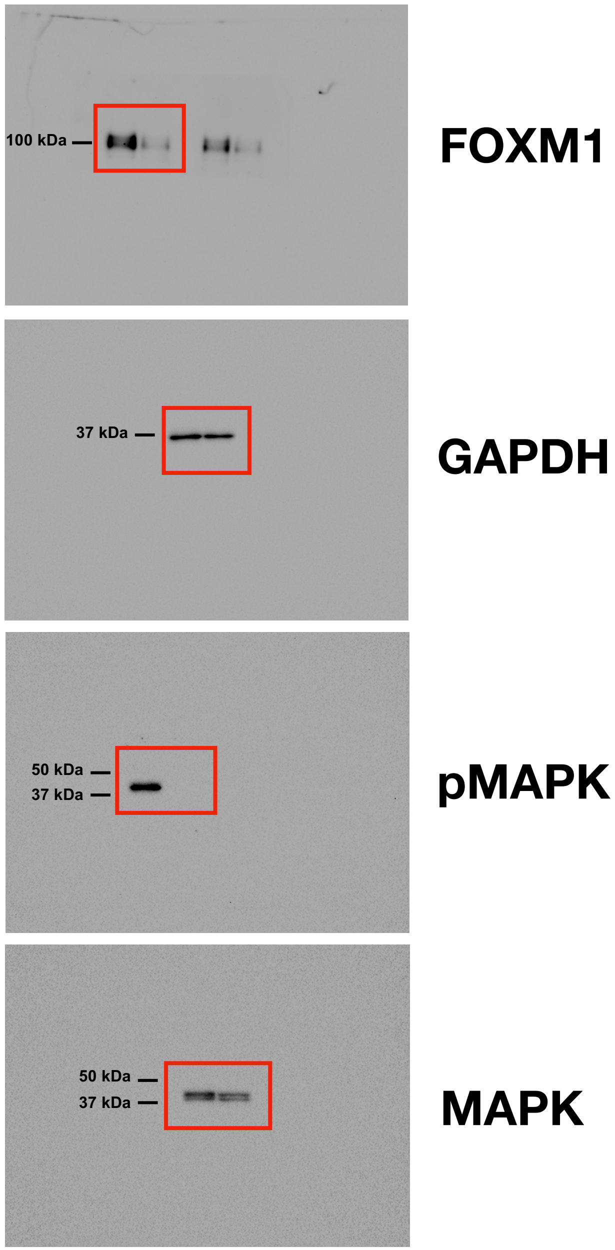

Supplementary Fig 4b

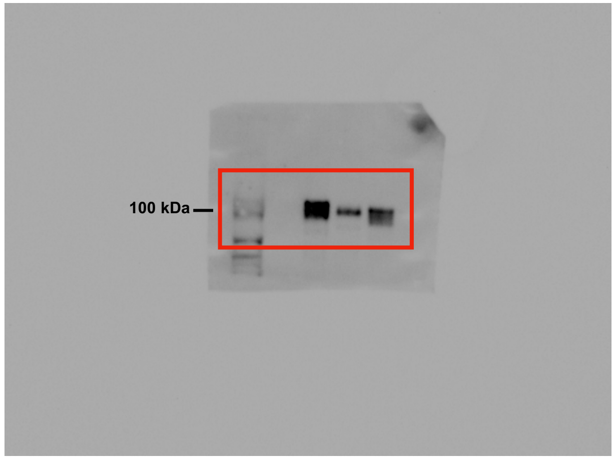

FOXM1

Fig 5c

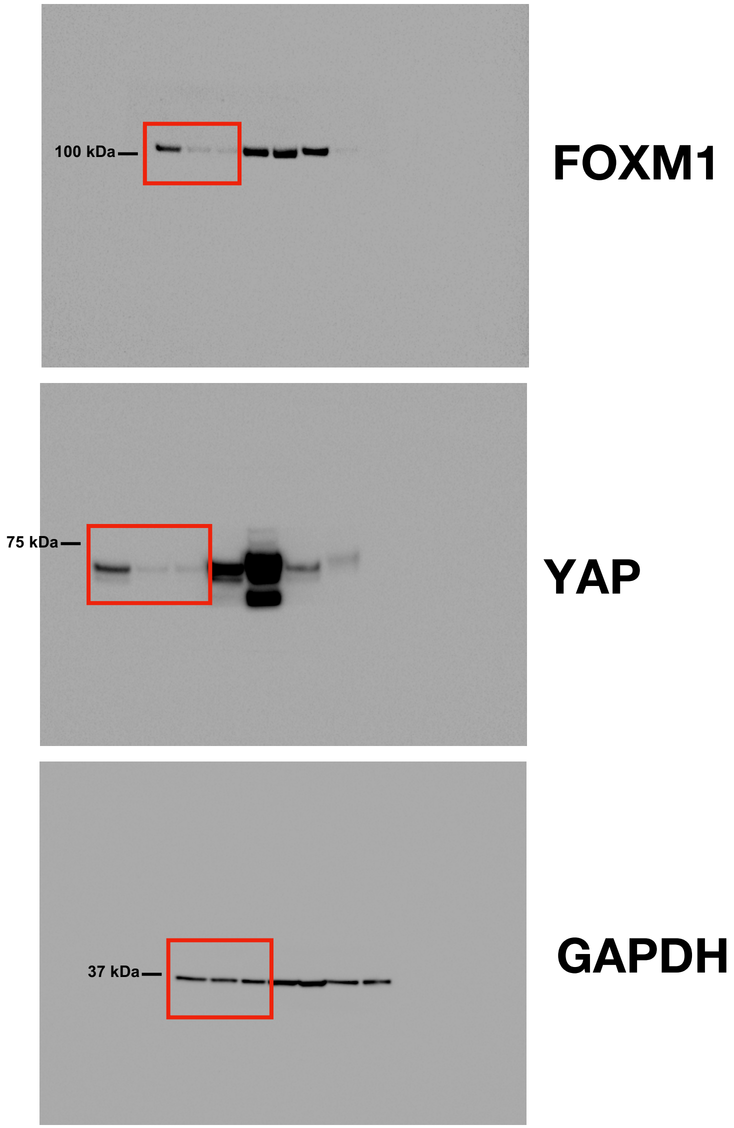

Fig 5d

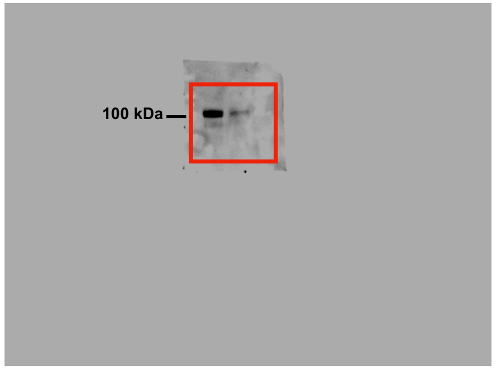

FOXM1

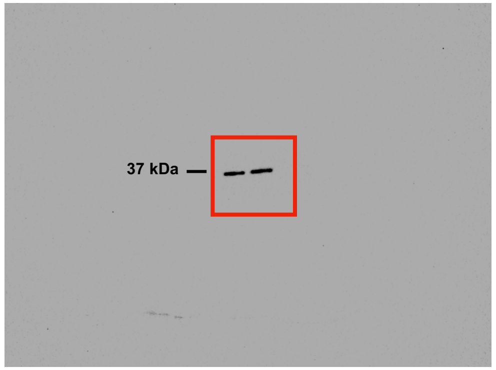

GAPDH

Fig. 6b

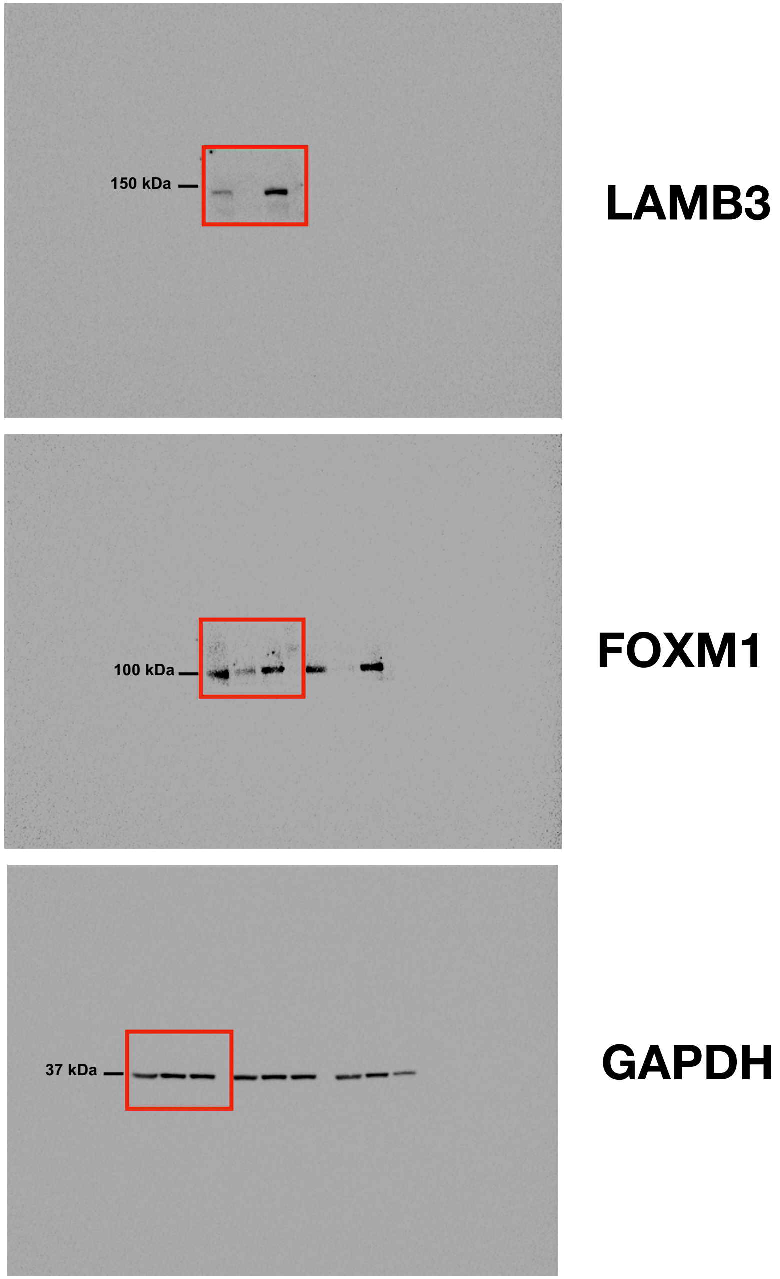

Fig 6c

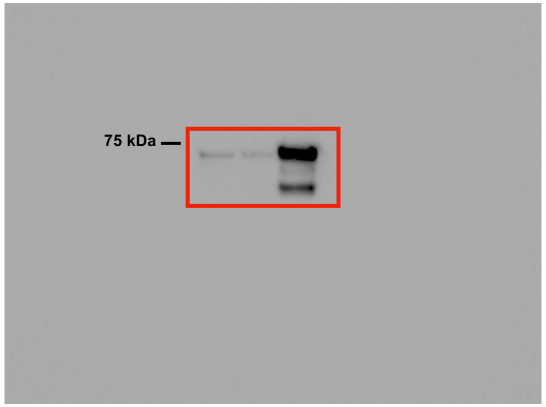

YAP

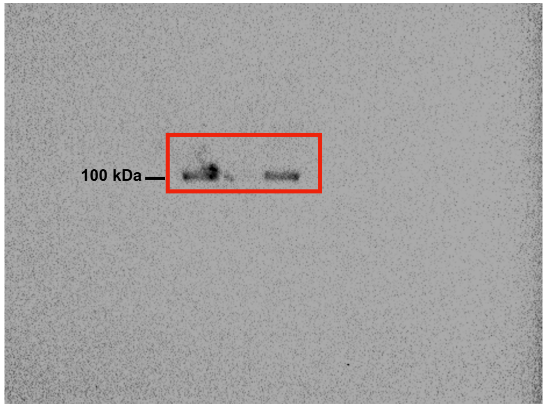

FOXM1

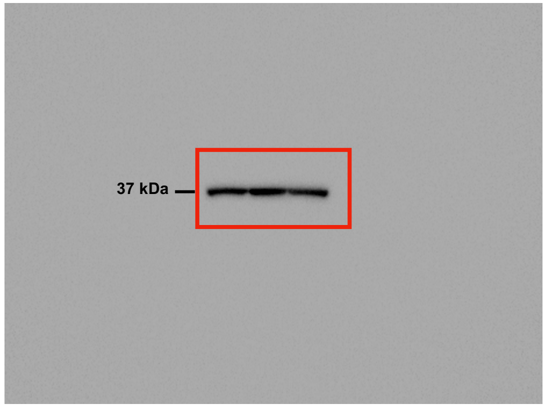

GAPDH

Fig 6d

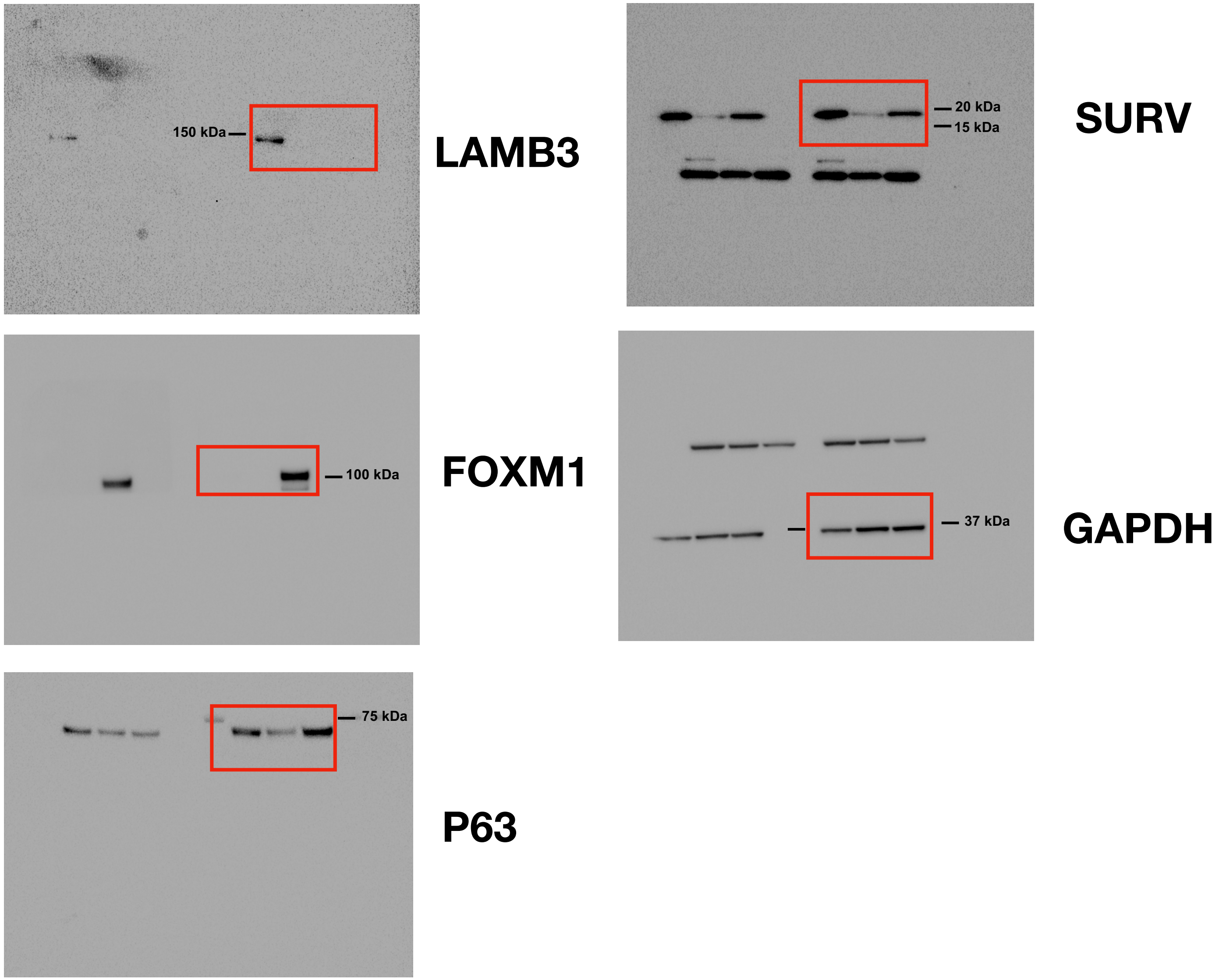
150 kDa
LAMB3
20 kDa
15 kDa
SURV
100 kDa
FOXM1
37 kDa
GAPDH
75 kDa
P63
